# Effect of Smoking on Lung Function Decline in a Retrospective Study of a Health Examination Population in Chinese Males

**DOI:** 10.3389/fmed.2022.843162

**Published:** 2023-01-06

**Authors:** Ting Tian, Xueqin Jiang, Rujie Qin, Yuqing Ding, Chengxiao Yu, Xin Xu, Ci Song

**Affiliations:** ^1^School of Public Health, Medical College of Yangzhou University, Yangzhou University, Yangzhou, China; ^2^Department of Geriatric Medicine, Huadong Sanatorium, Wuxi, China; ^3^Joyfulway Clinic, Fosun Health Co., Ltd., Shanghai, China; ^4^Department of Health Management, Huadong Sanatorium, Wuxi, China; ^5^Department of Medical Record Statistics, The Cancer Hospital of the University of Chinese Academy of Sciences (Zhejiang Cancer Hospital), Institute of Basic Medicine and Cancer (IBMC), Chinese Academy of Sciences, Hangzhou, China; ^6^Department of Epidemiology, Center for Global Health, School of Public Health, Nanjing Medical University, Nanjing, China; ^7^Jiangsu Key Lab of Cancer Biomarkers, Prevention and Treatment, Collaborative Innovation Center for Cancer Personalized Medicine, Nanjing Medical University, Nanjing, China

**Keywords:** smoking cessation, FEV_1_, FVC, FEV_1_/FVC, health examination

## Abstract

**Objective:**

China has established a goal of reducing adult smoking prevalence from 27.7% to 20% by 2030. Understanding the possible ongoing impairment in lung function in smokers, is critically important to encourage the populations to change their smoking behavior.

**Methods:**

A total of 14,273 males joined the health examination at Huadong Sanatorium from Jan 2012 to Dec 2019 were included. In cross-sectional analysis, we used multiple linear regression to evaluate the association between baseline lung function and smoking status. Then, 3,558 males who received ≥2 spirometry exams were analyzed in longitudinal study. Annual lung function decline was compared using mixed linear models adjusted for confounders.

**Results:**

In cross-sectional analysis, compared with never-smokers, decreases of −133.56 mL (95% CI: −167.27, −99.85) and −51.44 mL (−69.62, −33.26) in FEV_1_, −1.48% (−1.94, −1.02) and −1.29% (−1.53, −1.04) in FEV_1_/FVC were observed in former and current smokers. In longitudinal analysis, significant declines were observed in FEV_1_ [5.04 (2.30, 7.78) mL] and FEV_1_/FVC [0.09 (0.05, 0.13) %] in current smokers but not observed in former smokers after adjustment. Participants with long duration of smoking cessation had decelerate lung function than short duration. The annual decline rate of current smokers with high smoking intensity (≥30 cigarettes per day) was 13.80 and 14.17 times greater than that of never-smokers in FEV_1_ and FVC. Thus, early smoking cessation can slow down lung function decline trend for current smokers.

**Conclusions:**

The harms of current smoking on lung function emphasize the necessity of smoking cessation, especially for those with comorbidities.

## Introduction

Smoking is the major cause of premature death worldwide (1, 2). As the Global Adult Tobacco Survey (GATS) approximated in 2010, China is the largest producer and consumer of tobacco products in the world, with an estimation of 301 million current smokers (3–6). The current smoking prevalence among men was 52.9% and that among women was 2.4% in China (5). Such a high smoking rate of Chinese males shifts the negative effects on pulmonary health and accounts for nearly 20% of all-cause mortality during the past decades (7). The magnitude of tobacco related pulmonary disease has created a healthcare crisis in China (3).

Lung function is a critical measurement and early severity predictor for indicating cardio-pulmonary health (8). Decline of FEV_1_ indicates a higher risk of COPD (9); while the ratio of forced expiratory volume in 1 s (FEV_1_) to forced vital capacity (FVC), also known as FEV_1_/FVC, is the primary index of airflow limitation or airway obstruction (10). Current smoking was found associated with accelerated age-related FEV_1_ decline (11, 12). While one meta-analysis showed a homogeneity effect of current and former smoking on FEV_1_ decline (9). However, former smokers having changed the smoking habits for part of the period during which the betas were estimated may lead to the non-significant estimates in this meta-analysis (9). Furthermore, prior studies always focused on the association between smoking status and FEV_1_ decline among COPD or asthma population (13–15). Besides, the mentioned studies were mainly conducted in the developed countries, e.g., the United States, Swedish, UK et.al. In these countries, workplace smoking cessation (SC) intervention is effective in increasing quit rate and more cases were voluntary SC promotion (16). In contrast, Chinese populations were less likely to promote voluntary SC, most of them quit smoking due to smoking-related diseases. Thus, it is essential to evaluate the association between different smoking status and lung function decline in the Chinese population.

As of the Health China 2030 strategy, the government has established a goal of reducing adult smoking prevalence from 27.7 to 20% by 2030 (17). Challenges remain in accomplishing the goal. Understanding the possible ongoing impairment of smoking in lung function, is increasingly important, to encourage the voluntary SC promotion. Hence, we conducted this retrospective study to evaluate the association between smoking exposure and changes of lung function (i.e., FEV_1_, FVC and FEV_1_/FVC) among Chinese males with repeated measure of the indicators.

## Materials and Methods

### Data Source

We used data from Huadong Sanatorium health examination database (HSHED) between Jan 1, 2012 and Dec 31, 2019. Huadong Sanatorium (HS) is a municipal medical institution integrating convalescence, rehabilitation and health care, which providing personalized health management services for the entire society. Most participants taking health examination in HS are employees of various employers from Shanghai aged 15–95 years old. HSHED was established based on hospital information system (HIS) in 2003. All the results of examination were recorded in the HSHED.

We extracted data from participants who volunteered to receive basic health examination and additional spirometry exams in HS. A total of 22,051 participants took spirometry exams were included. Date when participants first underwent a spirometry exam in HS was set as baseline. Female participants, with low smoking rate (< 1%), were excluded, left 14,273 males to evaluate the association between lung function and smoking status in the cross-sectional analysis phase (Substudy 1, Supplementary Figure 1). In order to examine the longitudinal association of lung function annual changes with smoking status, we restricted to the participants with valid spirometry at two or more exams. Then, 3,558 males were included in the longitudinal analysis (Substudy 2, Supplementary Figure 1).

The approval of this study was obtained from ethics committees at Huadong Sanatorium (No. 2020-01). Anonymized and de-identified information were used for analyses, and therefore informed consent was not required.

### Measurements

Smoking status was self-reported as “never” “former” and “current” cigarettes smoking at each spirometry exam. Ever-smokers were defined as former and current smokers. In the cross-sectional analysis phase, all the 14,273 participants were divided into three groups according to baseline smoking status: never-smokers (*N* = 5,468), former smokers (*N* = 1,111), and current smokers (*N* = 7,694) (Table 1). In the longitudinal analysis phase, 3,268 participants reported smoking status unchanged across the follow-up period. These participants were classified as sustained never-smokers (*N* = 1,305), former smokers (*N* = 245), and current smokers (*N* = 1,718). Other 290 participants were classified as having variable smoking status (Supplementary Table 1).

**Table 1:** Baseline characteristics of 14,273 male participants according to smoking status in the cross-section analysis.

	**Overall**	**Never-smokers**	**Former smokers**	**Current smokers**	***P* ^c^**
	**(*N =* 14,273)**	**(*N =* 5,468)**	**(*N =* 1,111)**	**(*N =* 7,694)**	
**Age, years**	48.52 (11.30)	46.66 (12.84)	54.41 (10.22)	49.00 (9.86)	< 0.001^b^
< 50	7,551 (52.90%)	3,236 (59.18%)	360 (32.40%)	3,955 (51.40%)	< 0.001
≥50	6,722 (47.10%)	2,232 (40.82%)	751 (67.60%)	3,739 (48.60%)	
**Height, cm**	171.17 (5.95)	171.28 (6.05)	170.59 (5.93)	171.18 (5.87)	0.001^b^
**weight, kg**	73.91 (10.29)	73.64 (10.31)	74.24 (9.44)	74.06 (10.39)	0.006^b^
**Body-mass index, kg/m** ^**2**^	25.20 (3.06)	25.08 (3.07)	25.50 (2.86)	25.24 (3.07)	< 0.001^b^
Normal weight (18.5–24.9)	6,656 (46.63%)	2,649 (48.45%)	465 (41.85%)	3,542 (46.04%)	< 0.001
Underweight (< 18.5)	171 (1.20%)	70 (1.28%)	7 (0.63%)	94 (1.22%)	
Overweight (25–29.9)	6,565 (46.00%)	2,432 (44.48%)	571 (51.40%)	3,562 (46.30%)	
Obesity (≥30)	881 (6.17%)	317 (5.80%)	68 (6.12%)	496 (6.45%)	
**Alcohol consumption**
Never	3,759 (26.34%)	1,867 (34.14%)	233 (20.97%)	1,659 (21.56%)	< 0.001
Former	214 (1.50%)	49 (0.90%)	70 (6.30%)	95 (1.23%)	
Current	10,173 (71.27%)	3,494 (63.90%)	782 (70.39%)	5,897 (76.64%)	
Unknown	127 (0.89%)	58 (1.06%)	26 (2.34%)	43 (0.56%)	
**Smoking behavior**
Pack-years	24.54 (18.61)	-	23.02 (18.41)	24.69 (18.63)	0.181^a^
Cigarettes per day	17.99 (9.92)	-	18.58 (11.23)	17.94 (9.78)	0.903^b^
**Hypertension**	5,093 (35.68%)	1,805 (33.01%)	516 (46.44%)	2,772 (36.03%)	< 0.001
**Diabetes**	5,365 (37.59%)	2,002 (36.61%)	455 (40.95%)	2,908 (37.80%)	0.021
**Elevated TC (>5.2 mmol/L)**	5,576 (39.24%)	2,007 (36.89%)	446 (40.40%)	3,123 (40.74%)	< 0.001
**Elevated TG (>1.7 mmol/L)**	5,932 (41.75%)	1,835 (33.73%)	424 (38.41%)	3,673 (47.92%)	< 0.001
**Elevated total bilirubin (>24.20 umol/L)**	664 (4.67%)	334 (6.14%)	63 (5.71%)	267 (3.48%)	< 0.001
**Diagnosed clinical lung disease**
COPD	5 (0.04%)	0 (0.00%)	2 (0.18%)	3 (0.04%)	0.013
Asthma	76 (0.53%)	41 (0.75%)	14 (1.26%)	21 (0.27%)	< 0.001
Chronic bronchitis	158 (1.11%)	29 (0.53%)	20 (1.80%)	109 (1.42%)	< 0.001
Bronchiectasis	50 (0.35%)	31 (0.57%)	5 (0.45%)	14 (0.18%)	0.001
Emphysema	243 (1.70%)	19 (0.35%)	38 (3.42%)	186 (2.42%)	< 0.001
Bullae	184 (1.29%)	42 (0.77%)	21 (1.89%)	121 (1.57%)	< 0.001
Postoperative lung cancer	22 (0.15%)	9 (0.16%)	12 (1.08%)	1 (0.01%)	< 0.001

Data are n (%), mean (SD).

TC, total cholesterol; TG, triglycerides; COPD, chronic obstructive lung disease.

^a^One-way ANOVA test for the equal variances.

^b^Kruskal-Wallis test for the unequal variances.

^c^Chi-square test.

In this analysis, 65 participants missing detailed information of TC, TG, total bilirubin. Among ever-smokers, only 2,781 ever-smokers had detailed information of pack-years and 2,936 current smokers had detailed information of cigerattes consumptions.

Spirometry was performed using a MiniSpir spirometer at baseline and follow-up visits. A bronchodilator was not administered prior to spirometry. Lung function was measured with standardized protocols by the same equipment and acquired by the same investigators. To harmonize these data, we retrospectively did quality control checks according to the American Thoracic Society/European Respiratory Society 2005 standards, which define valid exams as two or more acceptable curves reproducible within 150 mL (18). Lung function outcomes were forced expiratory volume in one second (FEV_1_), forced vital capacity (FVC), and their ratio (FEV_1_/FVC). The Global Lung Function Initiative equations (19) were used to define lower limit of normal (LLN).

Diagnosed clinical lung disease was defined as self-reported physician diagnosis of COPD, asthma, chronic bronchitis, bronchiectasis, emphysema, bullae and postoperative lung cancer. Airflow limitation was defined as FEV_1_/FVC lower than the LLN, defined by the NHANES III reference equations (20). Restrictive pattern was defined as FEV_1_/FVC≥LLN and FVC < LLN (21).

### Statistical Analysis

#### Baseline Characteristics of the Participants

Demographic characteristics of the study participants according to baseline smoking status were calculated and compared among groups. Baseline characteristics were assessed by one-way ANOVA, Chi-squared and Kruskal-Wallis test. Analyses were performed separately for Substudy 1 and Substudy 2.

#### Relationship Between Smoking Status and Lung Function at Baseline and Follow-Up

Firstly, we examined the relationship between smoking status and lung function at participants' first visit using cross-sectional analysis. We evaluated the mean differences in the lung function across different smoking exposure by multiple linear regression analysis.

To further evaluate the decline rate of lung function among different smoking status, longitudinal analysis was then performed. In this analysis, linear mixed models were used to test associations with repeated measures of lung function.

#### Sensitivity Analysis

Analyses were repeated in the participants without prevalent lung disease, with variable smoking status or aged older than 30 years to minimize the potential confounding effect.

Methods of smoking details, clinical and laboratory assessments, multiple linear regression, and linear mixed models were provided in the Supplementary Material. Data were analyzed using STATA software version 13 (STATA Corp, College Station, TX, USA). Statistical significance was defined as a two-tailed *P* < 0.05.

## Results

### Substudy 1 Cross-Sectional Associations of Lung Function With Smoking Exposures Among 14,273 Male Participants at Baseline

In the cross-sectional phase, baseline characteristics are shown in Table 1. Most of them (90.31%) were aged 30–70 years old. Former smokers and currents smokers were older than never-smokers (*P* < 0.001). Mean cumulative cigarette exposure of former smokers and current smokers were 23.02 ± 18.41 and 24.69 ± 18.63 pack-years (PYs), respectively. Current smokers consumed an average of 17.94 ± 9.78 cigarettes per day. Former smokers were more likely to have an underlying disease at the first visit, i.e., hypertension (46.44%), diabetes (40.95%), elevated total bilirubin (5.71%) and lung diseases (10.08%), when compared with never smokers and current smokers (*P* < 0.001).

Multiple linear regression was used to evaluate the associations between smoking exposures with lung function (Table 2). After adjustment, ever-smoking was significantly related with lower FEV_1_ and FEV_1_/FVC at the first visit. Compared with never-smokers, current smokers had a −51.44 mL (95% CI: −69.62, −33.26, *P* < 0.001) decrease in FEV_1_ and a −1.29% (95% CI: −1.53, −1.04, *P* < 0.001) decrease in FEV_1_/FVC; and former smokers had an even lower level of lung function, with a −133.56 mL (95% CI: −167.27, −99.85, *P* < 0.001) decrease in FEV_1_ and a −1.48% (95% CI: −1.94, −1.02, *P* < 0.001) decrease in FEV_1_/FVC. For ever-smokers, greater cumulative cigarettes consumptions were associated with lower lung function, significantly when the cumulative pack-years exceeded to 20–30 or ≥30 PYs [Mean difference for FEV_1_: −101.70(−145.84, −57.57) and −143.22(−180.24, −106.20); FVC: −66.22(−120.95, −11.50) and −80.75(−126.66, −34.85); FEV_1_/FVC: −1.15(−1.75, −0.55) and −2.06(−2.57, −1.55) for 20–30 PYs and ≥30 PYs, when compared to never-smoking, Table 2]. For former smokers, longer durations of smoking cessation had a lower lung function [Mean difference for FEV_1_: −214.28 (−336.45, −92.12) vs. −135.75 (−192.29, −79.20); FVC: −213.93 (−365.52, −62.34) vs. −131.52 (−201.68, −61.36); FEV_1_/FVC: −1.42 (−3.08, 0.25) vs. −1.15 (−1.92, −0.38) for ≥10 and < 10 years cessation duration]. For current smokers, those with ≥10 cigarettes/day had significant FEV_1_ and FEV_1_/FVC decline compared with never-smokers [Mean difference for FEV_1_: −46.70 (−85.14, −8.26), −92.73 (−124.65, −60.82), −80.71 (−138.31, −23.12); FEV_1_/FVC: −0.61 (−1.13, −0.08), −0.94 (−1.37, −0.51), −1.73 (−2.51, −0.94) for 10–20, 20–30, and ≥30 cigarettes/day, respectively].

**Table 2:** Cross-sectional associations of lung function and different smoking status at baseline.

	**Number of** **participants**	**FEV**_**1**_ **(mL)**		**FVC (mL)**		**FEV**_**1**_ **/FVC (%)**	
		Mean Difference (95% CI)^*^	*P* ^*^	Mean Difference (95% CI)^*^	*P* ^*^	Mean Difference (95% CI)^*^	*P* ^*^
**Smoking status**							
Never-smokers	5,468	Ref		Ref		Ref	
Former smokers	1,111	−133.56 (−167.27, −99.85)	< 0.001	−102.10 (−143.94, −60.26)	< 0.001	−1.48 (−1.94, −1.02)	< 0.001
Current smokers	7,694	−51.44 (−69.62, −33.26)	< 0.001	1.75 (−20.81, 24.32)	0.879	−1.29 (−1.53, −1.04)	< 0.001
**Duration of smoking cessation**							
Never-smokers	5,468	Ref		Ref		Ref	
Former smokers, by duration of cessation							
≥10 years	68	−214.28 (−336.45, −92.12)	0.001	−213.93 (−365.52, −62.34)	0.006	−1.42 (−3.08, 0.25)	0.095
< 10 years	336	−135.75 (−192.29, −79.20)	< 0.001	−131.52 (−201.68, −61.36)	< 0.001	−1.15 (−1.92, −0.38)	0.003
**Cumulative cigarette consumption**							
Never-smokers	5,468	Ref		Ref		Ref	
Ever smokers to by pack-years							
< 10 pack-year	577	5.02 (−39.29, 49.32)	0.824	3.43 (−51.51, 58.37)	0.903	−0.03 (−0.63, 0.58)	0.933
10 to < 20 pack-years	672	−62.56 (−103.84, −21.27)	0.003	−75.88 (−127.08, −24.69)	0.004	−0.06 (−0.63, 0.50)	0.827
20 to < 30 pack-years	585	−101.70 (−145.84, −57.57)	< 0.001	−66.22 (−120.95, −11.50)	0.018	−1.15 (−1.75, −0.55)	< 0.001
≥30 pack-years	947	−143.22 (−180.24, −106.20)	< 0.001	−80.75 (−126.66, −34.85)	0.001	−2.06 (−2.57, −1.55)	< 0.001
**Current cigarette consumption**							
Never-smokers	5,468	Ref		Ref		Ref	
Current smokers to by cigarette per day							
< 10 cigarettes per day	326	−41.52 (−98.41, 15.36)	0.152	−41.99 (−112.87, 28.89)	0.246	−0.17 (−0.94, 0.60)	0.670
10 to < 20 cigarette per day	775	−46.70 (−85.14, −8.26)	0.017	−30.23 (−78.12, 17.67)	0.216	−0.61 (−1.13, −0.08)	0.023
20 to < 30 cigarette per day	1,251	−92.73 (−124.65, −60.82)	< 0.001	−66.51 (−106.28, −26.74)	0.001	−0.94 (−1.37, −0.51)	< 0.001
≥30 cigarette per day	327	−80.71 (−138.31, −23.12)	0.006	−14.84 (−86.61, 56.93)	0.685	−1.73 (−2.51, −0.94)	< 0.001

FEV_1_, forced expiratory volume in 1 s; FVC, forced vital capacity; CI, confidence interval.

Mean difference in spirometry measures in each category of smoking exposure and the reference category (never-smokers).

*Multivariable cross-sectional analyses were adjusted for baseline covariates: age (< 50/≥50), height, weight, BMI (normal/underweight/overweight/obesity), alcohol intake (never/former/current/unknown), hypertension (yes/no), diabetes (yes/no), elevated triglycerides (yes/no), elevated total cholesterol (yes/no), elevated total bilirubin (yes/no).

### Substudy 2 Longitudinal Associations Between Smoking Exposures and Lung Function Among 3,558 Male Participants

In the previous step, former smokers were observed with a lower level of lung function than that of never or current smokers. We further explored whether persistent smoking would accelerate the declines of lung function along with age. In the current longitudinal analyses, 3,558 male participants with ≥2 valid spirometry exams contributed 8,935 spirometry exams during follow-up. The baseline characteristics of 3,558 males in this longitudinal analysis was similar with those of participants in the cross-sectional analysis (Table 1 and Supplementary Table 2). The mean pack-years of current smokers was 25.18 ± 18.73, which was greater than that of former smokers (21.97 ± 15.44) before baseline. The mean values of FEV_1_, FVC, and FEV_1_/FVC for former smokers at baseline were lower than that of other participants. Compared with never-smokers and current smokers, former smokers were older, had a higher proportion of drinkers and more likely to have underlying diseases.

**Figure 1:**
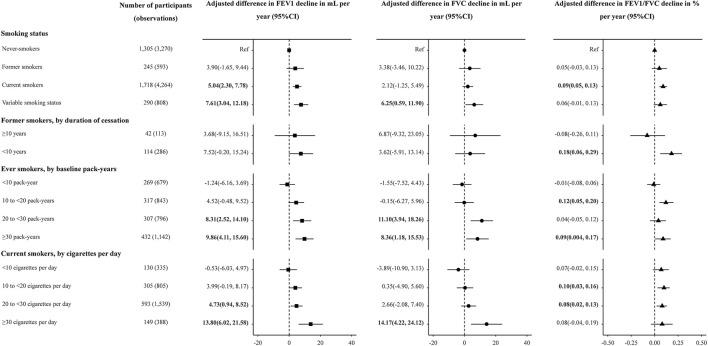
Adjusted association between smoking status, duration of smoking cessation, cumulative and current cigarette consumption, and lung function decline in the longitudinal analysis. FEV_1_, forced expiratory volume in 1 s; FVC, forced vital capacity; CI, confidence interval. Linear mixed models were used to test associations with repeated measures of FEV_1_, FVC and FEV_1_/FVC. Participants with variable smoking status were excluded from analyses of duration of smoking cessation and of cumulative and current cigarette consumption. Adjusted effect estimates for smoking exposures, relative to never-smoking, were generated with models adjusted for the smoking parameter, age, age^2^, height, weight, BMI, and alcohol consumption, hypertension, diabetes, TG, TC, total bilirubin at baseline. Multiplicative interactions with age were modeled for covariates. The effect estimate for smoking-exposure multiplied by age was interpreted as the association of the smoking exposure with annualized lung function decline.

Current smokers showed accelerated lung function decline compared with never-smokers. The unadjusted FEV_1_, FVC and FEV_1_/FVC decline among former smokers was 42.04 (36.77, 47.31), 47.36 (40.70, 54.03) mL and 0.16 (0.08, 0.24) % per year, compared to 33.99 (32.02, 35.97), 38.38 (35.90, 40.87) mL, and 0.09 (0.06, 0.11) % per year among never-smokers, and 39.26 (37.12, 41.40), 41.36 (38.55,44.17) mL, and 0.16 (0.14, 0.19)% per year among current smokers (Table 3). After adjusted for covariates, current smokers had an accelerated FEV_1_ decline of 5.04 ml (95% CI: 2.30, 7.78, *P* < 0.001) per year and FEV_1_/FVC decline of 0.09% (95% CI: 0.05, 0.13, *P* < 0.001) per year, when compared with never-smokers (Figure 1). Effect estimates were observed in participants with variable smoking status, with an accelerated FEV_1_ decline of 7.61 ml (95% CI: 3.04, 12.18, *P* = 0.001) per year and FVC decline of 6.25 ml (95%CI: 0.59, 11.90, *P* = 0.030) per year, compared to never-smokers. However, no significant estimates were analyzed for former smokers [FEV_1_, 3.90(−1.65, 9.44), *P* = 0.168; FVC, 3.38(−3.46, 10.22), *P* = 0.332; FEV_1_/FVC, 0.05(−0.03, 0.13), *P* = 0.224, Figure 1].

**Table 3:** Association between smoking status, duration of smoking cessation, cumulative and current cigarette consumption, and lung function decline in the longitudinal analysis.

	**Number of**	**Unadjusted FEV_**1**_**	**Unadjusted FVC**	**Unadjusted FEV_**1**_/FVC**
	**participants**	**decline in mL**	**decline in mL**	**decline in % per**
	**(observations)**	**per year (95% CI)**	**per year (95% CI)**	**year (95% CI)**
**Smoking status**				
Never-smokers	1,305 (3,270)	33.99 (32.02, 35.97)	38.38 (35.90, 40.87)	0.09 (0.06, 0.11)
Former smokers	245 (593)	42.04 (36.77, 47.31)	47.36 (40.70, 54.03)	0.16 (0.08, 0.24)
Current smokers	1,718 (4,264)	39.26 (37.12, 41.40)	41.36 (38.55, 44.17)	0.16 (0.14, 0.19)
Variable smoking status	290 (808)	43.82 (39.39, 48.26)	48.34 (42.46, 54.22)	0.13 (0.06, 0.19)
**Former smokers, by duration of cessation**				
≥10 years	42 (113)	42.49 (29.42, 55.57)	47.31 (29.67, 64.95)	0.09 (-0.09, 0.26)
< 10 years	114 (286)	42.25 (34.55, 49.96)	43.53 (33.44, 53.61)	0.24 (0.12, 0.36)
**Ever-smokers, by baseline pack-years**				
< 10 pack-year	269 (679)	31.99 (27.29, 36.69)	36.18 (30.34, 42.02)	0.06 (0.001, 0.12)
10 to < 20 pack-years	317 (843)	38.93 (33.91, 43.94)	39.43 (32.70, 46.17)	0.20 (0.13, 0.27)
20 to < 30 pack-years	307 (796)	44.09 (38.51, 49.67)	51.84 (44.65, 59.04)	0.13 (0.05, 0.21)
≥30 pack-years	432 (1,142)	47.48 (41.88, 53.09)	51.62 (44.35, 58.59)	0.20 (0.12, 0.29)
**Current smokers, by cigarettes per day**
< 10 cigarettes per day	130 (335)	30.52 (23.84, 37.19)	30.95 (23.11, 38.79)	0.12 (0.03, 0.21)
10 to < 20 cigarettes per day	305 (805)	36.78 (32.12, 41.44)	37.25 (31.32, 43.18)	0.19 (0.13, 0.25)
20 to < 30 cigarettes per day	593 (1,539)	40.70 (36.93, 44.46)	43.64 (38.74, 48.55)	0.17 (0.12, 0.22)
≥30 cigarettes per day	149 (388)	49.37 (40.64, 58.09)	55.45 (43.74, 67.16)	0.15 (0.03, 0.27)

FEV_1_, forced expiratory volume in 1 s; FVC, forced vital capacity; CI, confidence interval.

Unadjusted mean lung function decline was estimated from a model including only age as predictors.

Unadjusted models were created separately for each stratum of the primary exposures: smoking status, duration of smoking cessation for former smokers, baseline pack-years for ever smokers and cigarette exposure for current smokers.

For the former smokers, shorter durations of smoking cessation (< 10 years) were associated with more accelerated FEV_1_ decline than longer durations (≥10 years), when compared to never-smokers (3.68 vs. 7.52 mL, Figure 1). Compared with never-smokers, FEV_1_/FVC decline was accelerated by 0.18% per year (95% CI: 0.06, 0.29, *P* = 0.002) in former smokers with < 10 years of cessation, while the estimate was not obvious in former smokers with ≥10 years of cessation (*P* = 0.438) (Figure 1). Compared to never-smokers, decline was accelerated by 8.68 mL per year (*P* = 0.004) in FEV_1_ and by 0.10% per year (*P* = 0.029) in FEV_1_/FVC among 177 observed quitters (Supplementary Table 4).

For ever-smokers, the unadjusted estimates of declines in FEV_1_ accelerated with the increase of cumulative smoking pack-years (estimates for exposure with < 10, 10–20, 20–30, and ≥30 PYs were 31.99, 38.93, 44.09, and 47.48, respectively, Table 3). In the adjusted model, adjusted mean FEV_1_ decline accelerated with increased pack-years (estimates for exposure with < 10, 10–20, 20–30 and ≥30 PYs were −1.24, 4.52, 8.31, and 9.86, respectively, Figure 1). The adjusted effect estimate of FVC decline was significant but attenuated among participants with ≥20 PYs (estimates for exposure with 20–30 and ≥30 PYs were 11.10 and 8.36, respectively, Figure 1).

At the levels of current smoking intensity, current smokers with greater smoking intensity had more accelerate in FEV_1_ and FVC decline (Table 3 and Figure 1). The adjusted effect estimates of FEV_1_ decline for those smoking ≥30 cigarettes per day (13.80, 95% CI: 6.02, 21.58, *P* < 0.001) was 2.92 times greater than that for those smoking 20–30 cigarettes per day (4.73, 95% CI: 0.94, 8.52, *P* = 0.015). Compared with never-smokers, FVC decline was accelerated by 14.17 mL per year (95% CI: 4.22, 24.12) in current smokers with ≥30 cigarettes per day (Figure 1).

Although there was statistical evidence of effect modification by age, height and weight (*P* < 0.05), effect sizes did not change considerably across strata of age, height and weight (Supplementary Figure 2). As former smokers had a higher prevalence of hypertension and diabetes, we further explored the associations stratifying by baseline diagnosis of hypertension and diabetes. For never-smokers, participants with baseline hypertension and diabetes had more accelerated FEV_1_ decline than those without underlying diseases (*P* = 0.008, data not shown in Supplementary Figure 3). Among participants without hypertension and diabetes, current smokers had accelerated FEV_1_ decline compared to never-smokers after adjustment (*P* = 0.004, data not shown in Supplementary Figure 3), which can also show that current smoking was an independent risk factor for lung function. Compared with never-smokers without underlying hypertension and diabetes, FEV_1_ decreased more rapidly in heavy smokers (≥20 pack-years for ever-smokers and ≥20 cigarettes per day for current smokers) with underlying hypertension and diabetes (*P* = 0.032 and < 0.001, data not shown in Supplementary Figure 3).

### Sensitivity Analysis

After excluding the participants with prevalent lung diseases, the cross-sectional associations were slightly attenuated (Supplementary Table 3). The same longitudinal analyses were repeated among the participants without prevalent lung diseases (Supplementary Figure 4). The similar mean estimates were observed in these sensitivity analyses.

## Discussion

Our study has documented the cross-sectional and longitudinal associations of smoking exposure and lung function in a general male population in China. Compared with never-smokers, we found that ever-smokers had a worse lung function. Current smokers, if not quit, would have an accelerated decline of lung function than former and never smokers. Furthermore, smokers with comorbid conditions, such as hypertension, elevated triglycerides and elevated total cholesterol, should raise more concerns about their lung health.

Evidence of significant decline of lung function in smokers has been present (21, 22). The unadjusted mean decline in FEV_1_ in healthy male never-smokers in our study were similar to that in the European Community Respiratory Health Survey (22). In our cross-sectional study, former smokers had a worse lung function than never smokers, even than current smokers. An older age, with more comorbid conditions including hypertension, diabetes, and lung diseases in those former smokers may account for this phenomenon. In the longitudinal assessment, current cigarette smokers showed a more rapid decline in lung function than never-smokers and former smokers. Greater pack-years and cigarette consumptions have been associated with accelerated lung function decline in our study, which was consistent with previous study (23). Several previous studies have indicated that smoking cessation has a beneficial effect on FEV_1_ decline (12, 21, 24). Our findings showed that although the former smokers had worse lung function at baseline, their annual declines in lung function were approximately identical to those of never-smokers during the follow-up. All the results above reinforce the importance of smoking cessation.

Cigarette smoking leads to numerous pulmonary and systemic immunological changes (25). Previous studies have indicated that smoking increases the number of macrophages, neutrophils, eosinophils, and mast cells in the lung, and decreases the number of airway dendritic cells, and alters macrophage and neutrophil function (26, 27). These pathways of inflammation and immunity making the lung dysregulation have been observed to be associated with smoking-related lung function decline. Additionally, smoking can decelerate the lung function along with epigenetic alterations (28), airway hyper-responsiveness (29), mucous hypersecretion (30), and altered airway dimensions (31).

One strength of the present study was that we conducted two sub-studies to evaluate the association of smoking exposure and lung function among healthy subjects in China. In addition, the dynamic data of smoking status and lung function can be collected during the follow-up. However, several limitations of this study should not be ignored. Firstly, the sample size of participant received two or more spirometry exams was relatively short. Due to lack of standard questionnaire, part of the participants did not report the detailed information of smoking exposure such as duration of cessation, cigarette consumptions. Secondly, China is both the world's largest producer and consumer of tobacco products, with 52.9% of men and 2.4% of women being current smokers in 2010 (3, 4, 6). Regarding this situation, we only restricted male subjects to samples in this study. The number of former smokers was significantly less than never-smokers and current smokers. Thirdly, despite the large sample size, the included participants were limited in the single center. Further robust epidemiological evidence and functional study is urgently needed to better understand the biological mechanism of smoking exposure on lung function. Moreover, some potential confounders, such as physical activities, exercises and second-hand smoke exposure, cannot be collected.

In 2016, President Xi Jinping announced the Healthy China (HC2030) blueprint. According to the blueprint, a target to reduce the smoking rate among people ≥15 years of age to 20% by 2030 from the current 27.7% has been set (17, 32). To achieve this goal, more and more current smokers should participate in quitting smoking. Our data suggest that smoking cessation can slow the lung function decline even if the initial state of lung function is poor. It is essential for current smokers to quit smoking as soon as possible, especially for those with comorbidities.

## Conclusion

Our results therefore reinforce the view that acceleration of decline in lung function must be added to the long list of negative health consequences of smoking and that smoking cessation is the most effective means of harm reduction. Our findings about the harms of current smoking also raise concerns about lung health, especially for those with comorbid conditions, which can further encourage people to quit smoking.

## Data Availability Statement

The data analyzed in this study is subject to the following licenses/restrictions: Data of the present research is available from the corresponding author on reasonable request. Requests to access these datasets should be directed to 906921532@qq.com.

## Ethics Statement

The approval of this study was obtained from Ethics Committees at Huadong Sanatorium (No. 2020-01). Written informed consent for participation was not required for this study in accordance with the national legislation and the institutional requirements.

## Author Contributions

XJ: full access to all of the data in the study, takes responsibility for the integrity of the data, and the accuracy of the data analysis. TT and CS: concept and design and obtain funding. XJ and RQ: acquisition of data. TT and YD: drafting of the manuscript and statistical analysis. CS: critical revision of the manuscript for important intellectual content. CY and XX: administrative, technical, or material support. All authors contributed to the article and approved the submitted version.
