# Supplementary material for: Effect of Smoking on Lung Function Decline in a Retrospective Study of a Health Examination Population in Chinese Males
Source: Front Med (Lausanne). 2023 Jan 6;9:843162. doi: 10.3389/fmed.2022.843162 (PMC9853193; doi:10.3389/fmed.2022.843162)
Supplement: Supplementary file 1 [file Data_Sheet_1.docx]

***Supplementary Material***

Page 2 **Methods**

Page 4 **Table s1. Classification of smoking status over follow-up.**

Page 5 **Table s2. Baseline characteristics of 3,558 male participants according to smoking status in longitudinal analysis.**

Page 7 **Table s3. Cross-sectional association of lung function and different smoking status among participants without lung disease at baseline.**

Page 8 **Table s4. Associations between variable smoking status and lung function.**

Page 9 **Figure s1. Diagram plot.**

Page 9 Figure s2. Associations between smoking status and lung function decline (mL/year), stratified by age, height and weight in the longitudinal analysis.

Page 9 **Figure s3. Associations between smoking exposures and FEV1 decline (mL/year), stratified by baseline hypertension and diabetes in the longitudinal analysis.**

Page 9 **Figure s4. Longitudinal association between smoking status, duration of smoking cessation, cumulative and current cigarette consumption, and lung function decline among participants without prevalent lung diseases.**

**Methods**

**Smoking details**

In addition, the detailed information on the duration of smoking cessation, cumulative cigarette exposure and current cigarette exposure were collected. Duration of smoking cessation of the sustained former smokers were categorized at thresholds of less 10 years and 10 or more years. For participants in the cross-sectional analysis phase, the duration of smoking cessation was calculated as time from the quit date to the baseline spirometry exam; for participants in the longitudinal analysis phase, the duration of smoking cessation was calculated as time from the quit date to date of the last spirometry exam. Cumulative cigarette exposure was defined among sustained former smokers and current smokers in terms of pack-years (PYs), which were calculated at baseline as average cigarettes per day multiplied by years smoked and categorized at thresholds of 10, 20 and 30 PYs. Current cigarette exposure was defined among sustained current smokers by time-variant number of cigarettes per day, which was categorized into 4 groups (<10, 10 to <20, 20 to <30 and ≥30 cigarettes per day).

**Clinical and laboratory assessments**

Demographic characteristics, including age, smoking exposures, alcohol consumption and medical history (hypertension and diabetes) were self-reported by participants. Body mass index (BMI) was calculated by weight (kilogram)/height^2^ (square meter) for each participant. Blood pressure was recorded by trained nurses with an electronic sphygmomanometer after at least 5 min of seated rest. Hypertension was defined as systolic blood pressure (SBP) ≥140 mmHg, diastolic blood pressure (DBP) ≥90 mmHg, self-reported history of hypertension.^1^ Diabetes were defined as a self-reported history of diabetes, and/or glycated hemoglobin (HbA1c) ≥6.5%, or fasting blood glucose (FBG) ≥7.0 mmol/L.^2,3^

Blood samples were taken from participants after at least a 10-h fast and analyzed at the Laboratory Medicine Department at HS. Serum levels of FBG, total cholesterol (TC), triglycerides (TG), total bilirubin for each participant were detected by an automatic biochemical analyzer, with the reference value of 3.6-6.1mmol/L, 3.2-5.2 mmol/L, 0.5-1.7 mmol/L and 5.13-24.20 umol/L respectively.

**Multiple linear regression**

Multiple linear regression analyses were performed with adjustment for age(<50 / ≥50), height, weight, BMI (normal weight/underweight/overweight/obesity), alcohol intake (never/former/ current/unknown), hypertension (yes/no), diabetes (yes/no), elevated TG (yes/no), elevated TC (yes/no), elevated total bilirubin (yes/no). Missing values for categorical variables were coded as such and were not excluded from the analyses.

**Linear mixed models**

Linear mixed models were used to test associations with repeated measures of FEV_1_, FVC and FEV_1_/FVC. Because of the age-dependence of lung function and left truncation of data, the time scale for analyses was defined as age at exam. Random intercept and time effects were included at the individual level to differentiate between- and within-individual variation. Unadjusted mean lung function decline was estimated from a model including only age as predictors. Unadjusted models were created separately for each stratum of the primary exposures: smoking status, duration of smoking cessation for former smokers, pack-years for ever-smokers and cigarette exposure for current smokers. Adjusted effect estimates for smoking exposures, relative to never-smoking, were generated with models adjusted for a priori selected baseline factors and precision variables. These covariates were age, age-squared, height, weight, BMI, alcohol intake, hypertension, diabetes, TG, TC, total bilirubin. To account for potential confounding of associations with lung function slope, multiplicative interactions with age were modelled for all other covariates. The effect estimate for smoking-exposure multiplied by age was interpreted as the association of the smoking exposure with annualized lung function decline. Stratified by baseline hypertension, diabetes, TC and TG, linear mixed models were also used to evaluate the associations between smoking exposures and FEV_1_ decline.

**Reference**

1. James PA, Oparil S, Carter BL, et al. 2014 evidence-based guideline for the management of high blood pressure in adults: report from the panel members appointed to the Eighth Joint National Committee (JNC 8). *JAMA*.2014;311(5):507-20.

2. American Diabetes A. 2. Classification and Diagnosis of Diabetes: Standards of Medical Care in Diabetes-2020. *Diabetes Care*.2020;43(Suppl 1):S14-S31.

3. American Diabetes A. 2. Classification and Diagnosis of Diabetes. *Diabetes Care*.2016;39 Suppl 1:S13-22.

**Table s1. Classification of smoking status over follow-up.**

| **Smoking trajectory** | **No. participants** | **Percent** | **Average baseline age** |
| --- | --- | --- | --- |
| Never-smoker (never-smoker throughout) | 1,305 | 36.68% | 48.61(12.49) |
| Former smoker (former smoker throughout) | 245 | 6.89% | 56.46(10.29) |
| Current smoker (current smoker throughout) | 1,718 | 48.29% | 50.17(9.48) |
| Variable status |  |  |  |
| Observed quitter (from current to former smoker) | 177 | 4.97% | 53.42(9.85) |
| Started smoking (from never to current smoker) | 38 | 1.07% | 44.08(12.08) |
| Started and quit smoking (from never, to current, to former smoker) | 33 | 0.93% | 54.37(9.87) |
| Inconsistent transitions of smoking status (no standard pattern) | 42 | 1.19% | 49.83(8.65) |

**Table s2. Baseline characteristics of 3,558 male participants according to smoking status in longitudinal analysis.**

|  | **Overall**  **(N=3,558)** | **Never-smokers (N=1,305)** | **Former smokers (N=245)** | **Current smokers (N=1,718)** | **Variable smoking status (N=290)** | ***P* value^c^** |
| --- | --- | --- | --- | --- | --- | --- |
| **Age, years** | 48.69(10.89) | 46.93(12.26) | 55.16(10.18) | 48.80(9.38) | 50.43(10.86) | <0.001^b^ |
| <50 | 1,856(52.16%) | 753(57.70%) | 74(30.20%) | 890(51.80%) | 139(47.93%) | <0.001 |
| ≥50 | 1,702(47.84%) | 552(42.30%) | 171(69.80%) | 828(48.20%) | 151(52.07%) |  |
| **Height, cm** | 171.22(5.88) | 171.23(5.87) | 170.57(5.44) | 171.27(5.87) | 171.36(6.28) | 0.345^a^ |
| **weight, kg** | 73.73(9.97) | 73.21(9.89) | 73.16(9.44) | 74.21(10.08) | 73.77(9.96) | 0.040^a^ |
| **Body-mass index, kg/m^2^** | 25.13(2.99) | 24.95(2.98) | 25.15(3.01) | 25.27(3.00) | 25.10(2.92) | 0.033^a^ |
| Normal (18.5-24.9) | 1,693(47.58%) | 642(49.20%) | 109(44.49%) | 798(46.45) | 144(49.66%) | 0.160 |
| Underweight (<18.5) | 28(0.79%) | 11(0.84%) | 5(2.04%) | 11(0.64%) | 1(0.34%) |  |
| Overweight (25-29.9) | 1,653(46.46%) | 598(45.82%) | 117(47.76%) | 808(47.03%) | 130(4.83%) |  |
| Obesity (≥30) | 184(5.17%) | 54(4.14%) | 14(5.71%) | 101(5.88%) | 15(5.17%) |  |
| **Alcohol consumption** |  |  |  |  |  |  |
| Never | 952(26.76%) | 477(36.55%) | 50(20.41%) | 359(20.90%) | 66(22.76%) | <0.001 |
| Former | 59(1.66%) | 13(1.00%) | 16(6.53%) | 28(1.63%) | 2(0.69%) |  |
| Current | 2,521(70.85%) | 805(61.69%) | 174(71.02%) | 1,322(76.95%) | 220(75.86%) |  |
| Unknown | 26(0.73%) | 10(0.77%) | 5(2.04%) | 9(0.52%) | 2(0.69%) |  |
| **Smoking behavior** |  |  |  |  |  |  |
| Pack-years | 24.81(18.62) | - | 21.97(15.44) | 25.18(18.73) | 22.74(20.29) | 0.097^b^ |
| Cigarettes per day | 18.33(10.06) | - | 18.86(10.79) | 18.44(9.85) | 16.07(12.00) | 0.024^b^ |
| **Hypertension** | 1,257(35.33%) | 430(32.95%) | 108(44.08%) | 601(34.98%) | 118(40.69%) | 0.002 |
| **Diabetes** | 1,139(32.01%) | 387(29.66%) | 84(34.29%) | 582(33.88%) | 86(29.66%) | 0.060 |
| **Elevated TC (>5.2mmol/L)** | 1,376(38.73%) | 476(36.53%) | 89(36.48%) | 697(40.62%) | 114(39.31%) | 0.122 |
| **Elevate TG (>1.7mmol/L)** | 1,431(40.28%) | 444(34.08%) | 73(29.92%) | 798(46.50%) | 116(40.00%) | <0.001 |
| **Elevated** **total bilirubin(>24.20umol/L)** | 165(4.64%) | 80(6.14%) | 17(6.97%) | 59(3.44%) | 9(3.10%) | 0.001 |
| **Diagnosed clinical lung disease** |  |  |  |  |  |  |
| COPD | 1(0.03%) | 1(0.08%) | 0 | 0 | 0 | 0.631 |
| Asthma | 23(0.65%) | 10(0.77%) | 4(1.63%) | 8(0.47%) | 1(0.34%) | 0.152 |
| Chronic bronchitis | 25(0.70%) | 10(0.77%) | 1(0.41%) | 12(0.70%) | 2(0.69%) | 0.944 |
| Bronchiectasis | 11(0.31%) | 10(0.77%) | 0 | 1(0.06%) | 0 | 0.003 |
| Emphysema | 44(1.24%) | 4(0.31%) | 6(2.45%) | 28(1.63%) | 6(2.07%) | 0.001 |
| Bullae | 26(0.73%) | 5(0.38%) | 3(1.22%) | 14(0.81%) | 4(1.38%) | 0.183 |
| Postoperative lung cancer | 7(0.20%) | 3(0.23%) | 4(1.63%) | 0 | 0 | <0.001 |
| **Lung function** |  |  |  |  |  |  |
| FEV_1_, L | 3.25(0.64) | 3.30(0.65) | 3.02(0.66) | 3.24(0.62) | 3.21(0.66) | <0.001^a^ |
| FVC, L | 4.05(0.78) | 4.08(0.79) | 3.86(0.84) | 4.06(0.76) | 4.03(0.81) | <0.001^a^ |
| FEV_1_/FVC, % | 80.27(7.27) | 81.11(7.31) | 78.46(7.89) | 79.93(7.02) | 80.07(7.54) | <0.001^b^ |
| Airflow limitation | 326(13.12%) | 105(11.43%) | 26(15.95%) | 165(13.66%) | 30(15.38%) | 0.200 |
| Restrictive pattern | 1,073(33.20%) | 386(32.17%) | 82(37.44%) | 510(32.84%) | 95(36.54%) | 0.289 |
| **Number of spirometry exams** |  |  |  |  |  |  |
| 2 | 2,352 | 862 | 167 | 1,171 | 152 | - |
| 3 | 758 | 276 | 60 | 344 | 78 | - |
| 4 | 314 | 123 | 12 | 140 | 39 | - |
| ≥5 | 134 | 44 | 6 | 63 | 21 | - |
| **Number of spirometry observations** | 8,935 | 3,270 | 593 | 4,264 | 808 | - |

Data are n (%), mean (SD).

TC = total cholesterol; TG = triglycerides; COPD = chronic obstructive lung disease; FEV_1_= forced expiratory volume in 1 s; FVC = forced vital capacity;

^a^ One-way ANOVA test for the equal variances

^b^ Kruskal-Wallis test for the unequal variances

^c^ Chi-square test

In this analysis, 5 participants missing detailed information of TC, TG, total bilirubin. Among ever-smokers, only 1,325 ever-smokers had detailed information of pack-years and 1,177 current smokers had detailed information of cigerattes consumptions.

**Table s3. Cross-sectional association of lung function and different smoking status among participants without lung disease at baseline.**

|  | **Number of participants** | **FEV_1_** | |  | **FVC** | |  | **FEV_1_/FVC** | |
| --- | --- | --- | --- | --- | --- | --- | --- | --- | --- |
|  |  | **Mean Difference**  **(95% CI)*** | ***P**** |  | **Mean Difference**  **(95% CI)*** | ***P**** |  | **Mean Difference**  **(95% CI)*** | ***P**** |
| **Smoking status** |  |  |  |  |  |  |  |  |  |
| Never-smokers | 5,309 | Ref |  |  | Ref |  |  | Ref |  |
| Former smokers | 1,015 | -105.50(-139.56, -71.45) | <0.001 |  | -85.82(-128.90, -42.74) | <0.001 |  | -0.94(-1.39, -0.48) | <0.001 |
| Current smokers | 7,294 | -51.28(-69.29, -33.28) | <0.001 |  | -2.38(-25.16, 20.39) | 0.837 |  | -1.21(-1.45, -0.97) | <0.001 |
| **Duration of smoking cessation** |  |  |  |  |  |  |  |  |  |
| Never-smokers | 5,309 | Ref |  |  | Ref |  |  | Ref |  |
| Former smokers, by duration of cessation |  |  |  |  |  |  |  |  |  |
| ≥10 years | 61 | -130.16(-255.63, -4.70) | 0.042 |  | -125.15(-283.59, 33.30) | 0.122 |  | -0.80(-2.48, 0.87) | 0.347 |
| <10 years | 293 | -93.18(-151.89, -34.47) | 0.002 |  | -115.25(-189.38, -41.11) | 0.002 |  | -0.17(-0.95, 0.62) | 0.677 |
| Current smokers | 7,294 | -50.08(-68.08, -32.09) | <0.001 |  | -0.93(-23.66, 21.80) | 0.936 |  | -1.21(-1.45, -0.97) | <0.001 |
| **Cumulative cigarette consumption** |  |  |  |  |  |  |  |  |  |
| Never-smokers | 5,309 | Ref |  |  | Ref |  |  | Ref |  |
| Ever smokers, by pack-years |  |  |  |  |  |  |  |  |  |
| <10 pack-year | 560 | 5.95(-37.51, 49.41) | 0.788 |  | 0.23(-54.90, 55.35) | 0.994 |  | 0.05(-0.52, 0.63) | 0.860 |
| 10 to <20 pack-years | 620 | -62.13(-103.54, -20.72) | 0.003 |  | -82.84(-135.37, -30.31) | 0.002 |  | 0.11(-0.44, 0.66) | 0.693 |
| 20 to <30 pack-years | 526 | -87.61(-132.43, -42.79) | <0.001 |  | -73.30(-130.15, -16.45) | 0.012 |  | -0.64(-1.23, -0.04) | 0.036 |
| ≥30 pack-years | 798 | -114.06(-152.47, -75.65) | <0.001 |  | -67.08(-115.81, -18.36) | 0.007 |  | -1.45(-1.96, -0.94) | <0.001 |
| **Current cigarette consumption** |  |  |  |  |  |  |  |  |  |
| Never-smokers | 5,309 | Ref |  |  | Ref |  |  | Ref |  |
| Current smokers, by cigarette per day |  |  |  |  |  |  |  |  |  |
| <10 cigarettes per day | 312 | -42.84(-99.24, 13.57) | 0.137 |  | -44.74(-116.48, 27.00) | 0.222 |  | -0.19(-0.94, 0.56) | 0.619 |
| 10 to <20 cigarette per day | 717 | -39.64(-78.30, -0.98) | 0.044 |  | -35.61(-84.79, 13.56) | 0.156 |  | -0.30(-0.82, 0.21) | 0.244 |
| 20 to <30 cigarette per day | 1,119 | -79.06(-111.52, -46.59) | <0.001 |  | -62.68(-103.97, -21.39) | 0.003 |  | -0.62(-1.05, -0.19) | 0.005 |
| ≥30 cigarette per day | 296 | -89.79(-148.44, -31.14) | 0.003 |  | -45.19(-119.79, 29.40) | 0.235 |  | -1.40(-2.18, -0.62) | <0.001 |

FEV_1_= forced expiratory volume in 1 s; FVC = forced vital capacity; CI = confidence interval.

Mean difference in spirometry measures in each category of smoking exposure and the reference category (never-smokers).

*Multivariable cross-sectional analyses were adjusted for baseline covariates: age (<50/≥50), height, weight, BMI (normal/underweight/overweight/obesity), alcohol intake (never/former/current/unknown), hypertension (yes/no), diabetes (yes/no), elevated TG (yes/no), elevated TC (yes/no), elevated total bilirubin (yes/no).

**Table s4. Associations between variable smoking status and lung function.**

|  | **Number of participants  (observations)** | **FEV_1_** | | |  | **FVC** | | |  | **FEV_1_/FVC** | | |
| --- | --- | --- | --- | --- | --- | --- | --- | --- | --- | --- | --- | --- |
|  |  | **Unadjusted FEV_1_ decline in mL per year (95% CI)** | **Adjusted difference in FEV1 decline in mL per year (95% CI)** | ***P*** |  | **Unadjusted FVC decline in mL per year (95% CI)** | **Adjusted difference in FVC decline in mL per year (95% CI)** | ***P*** |  | **Unadjusted FEV_1_/FVC decline per year (95% CI)** | **Adjusted difference in FEV_1_/FVC decline per year (95% CI)** | ***P*** |
| ***All participants*** |  |  |  |  |  |  |  |  |  |  |  |  |
| Never-smokers | 1,305(3,270) | 33.99(32.02, 35.97) | Ref |  |  | 38.38(35.90, 40.87) | Ref |  |  | 0.09(0.06, 0.11) | Ref |  |
| Observed quitter | 177(491) | 46.15(40.14, 52.17) | **8.68(2.83, 14.54)** | **0.004** |  | 49.71(41.59, 57.83) | 5.45(-1.84, 12.74) | 0.143 |  | 0.15(0.06, 0.24) | **0.10(0.0, 0.18)** | **0.029** |
| Started smoking | 38(108) | 41.83(30.16, 53.50) | 7.13(-3.61, 17.87) | 0.193 |  | 45.08(29.89, 60.26) | 6.11(-7.10, 19.32) | 0.364 |  | 0.14(0.01, 0.26) | 0.08(-0.08, 0.24) | 0.339 |
| Started and quit smoking | 33(95) | 44.34(30.83, 57.85) | 6.08(-6.70, 18.87) | 0.351 |  | 56.76(38.65, 74.88) | 11.06(-4.81, 26.93) | 0.172 |  | 0.01(-0.18, 0.20) | -0.09(-0.28, 0.10) | 0.367 |
| Inconsistent transitions of smoking status | 42(114) | 42.42(30.72, 54.11) | 6.84(-6.36, 20.04) | 0.310 |  | 52.61(38.25, 66.97) | 10.72(-5.72, 27.16) | 0.201 |  | 0.04(-0.11, 0.20) | -0.04(-0.24, 0.15) | 0.674 |
|  |  |  |  |  |  |  |  |  |  |  |  |  |
| ***Participants without prevalent lung diseases*** |  |  |  |  |  |  |  |  |  |  |  |  |
| Never-smokers | 1,266(3,169) | 33.15(31.22, 35.08) | Ref |  |  | 37.62(35.12, 40.11) | Ref |  |  | 0.07(0.05, 0.09) | Ref |  |
| Observed quitter | 170(474) | 45.70(39.62, 51.77) | **9.06(3.26, 14.86)** | **0.002** |  | 49.69(41.32, 58.06) | 6.42(-0.95, 13.80) | 0.088 |  | 0.14(0.05, 0.23) | **0.10(0.02, 0.18)** | **0.018** |
| Started smoking | 38(108) | 41.83(30.16, 53.50) | 7.98(-2.44, 18.39) | 0.133 |  | 45.08(29.89, 60.26) | 6.58(-6.52, 19.67) | 0.325 |  | 0.14(0.01, 0.26) | 0.09(-0.06, 0.24) | 0.221 |
| Started and quit smoking | 32(93) | 46.21(32.45, 59.97) | 8.12(-4.62, 20.87) | 0.212 |  | 59.24(40.97, 77.51) | 12.90(-3.28, 29.08) | 0.118 |  | 0.02(-0.18, 0.21) | -0.05(-0.23, 0.13) | 0.610 |
| Inconsistent transitions of smoking status | 40(109) | 40.60(28.32, 52.87) | 7.27(-5.99, 20.53) | 0.282 |  | 51.03(36.03, 66.03) | 10.67(-6.15, 27.49) | 0.214 |  | 0.05(-0.11, 0.22) | -0.01(-0.20, 0.18) | 0.950 |

FEV_1_= forced expiratory volume in 1 s; FVC = forced vital capacity; CI = confidence interval.

Linear mixed models were used to test associations with repeated measures of FEV_1_, FVC and FEV_1_/FVC.

Unadjusted model was estimated from a model including only age as predictor and was done separately for each stratum of the primary exposure.

Adjusted effect estimates for smoking exposures, relative to never-smoking, were generated with models adjusted for the smoking parameter, age, age^2^, height, weight, BMI, and alcohol consumption, hypertension, diabetes, TG, TC, total bilirubin at baseline. Multiplicative interactions with age were modelled for covariates.

**Figure s1. Diagram plot.**

**Figure s2. Associations between smoking status and lung function decline (mL/year), stratified by age, height and weight in the longitudinal analysis.** Linear mixed models were used to test associations with repeated measures of FEV_1_, FVC and FEV_1_/FVC. Adjusted effect estimates for smoking exposures, relative to never-smoking, were generated with models adjusted for the smoking parameter, age, age^2^, height, weight, BMI, and alcohol consumption, TG, TC, total bilirubin at baseline. Multiplicative interactions with age were modelled for covariates. The effect estimate for smoking-exposure multiplied by age was interpreted as the association of the smoking exposure with annualized lung function decline. Participants with variable smoking status were excluded from analyses. The median values of height and weight were 171 cm and 73 kg.

**Figure s3. Associations between smoking exposures and FEV_1_ decline (mL/year), stratified by baseline hypertension and diabetes in the longitudinal analysis.** Linear mixed models were used to test associations with repeated measures of FEV_1_. Adjusted effect estimates for smoking exposures, relative to never-smoking, were generated with models adjusted for the smoking parameter, age, age^2^, height, weight, BMI, and alcohol consumption, TG, TC, total bilirubin at baseline. Multiplicative interactions with age were modelled for covariates. The effect estimate for smoking-exposure multiplied by age was interpreted as the association of the smoking exposure with annualized lung function decline. Participants with variable smoking status were excluded from analyses. Ever-smokers included former smokers and current smokers. Referent group was never-smokers without hypertension and diabetes at baseline. Hypertension and diabetes were baseline status.

**Figure s4. Longitudinal association between smoking status, duration of smoking cessation, cumulative and current cigarette consumption, and lung function decline among participants without prevalent lung diseases.** FEV_1_= forced expiratory volume in 1 s; FVC = forced vital capacity; CI = confidence interval. Linear mixed models were used to test associations with repeated measures of FEV_1_, FVC and FEV1/FVC. Participants with variable smoking status were excluded from analyses of duration of smoking cessation and of cumulative and current cigarette consumption. Unadjusted model was estimated from a model including only age as predictor and was done separately for each stratum of the primary exposure. Adjusted effect estimates for smoking exposures, relative to never-smoking, were generated with models adjusted for the smoking parameter, age, age^2^, height, weight, BMI, and alcohol consumption, hypertension, diabetes, TG, TC, total bilirubin at baseline. Multiplicative interactions with age were modelled for covariates. The effect estimate for smoking-exposure multiplied by age was interpreted as the association of the smoking exposure with annualized lung function decline.
