# Supplementary figures and images for: Effect of Smoking on Lung Function Decline in a Retrospective Study of a Health Examination Population in Chinese Males
Source: Front Med (Lausanne). 2023 Jan 6;9:843162. doi: 10.3389/fmed.2022.843162 (PMC9853193; doi:10.3389/fmed.2022.843162)

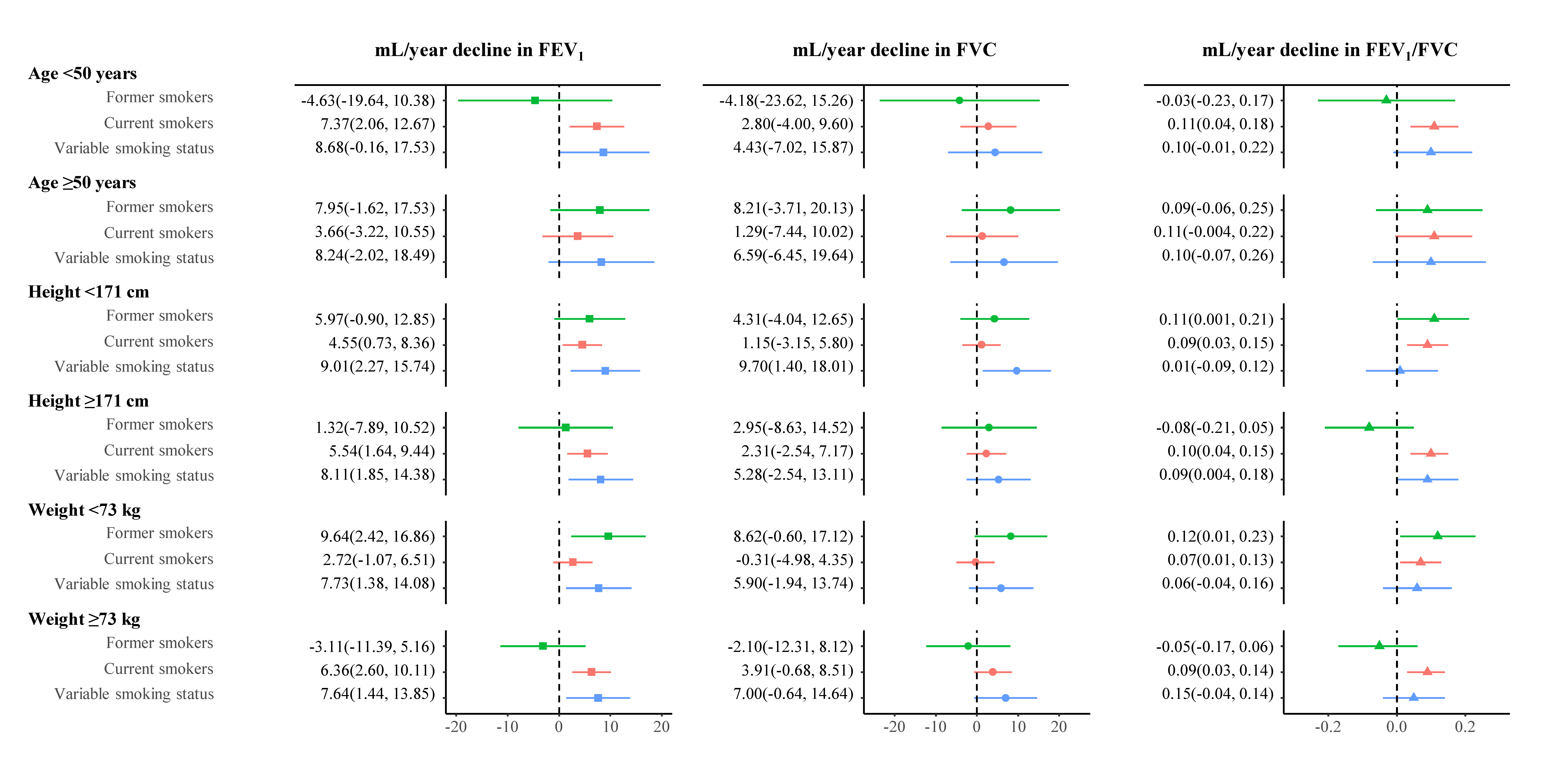

Supplement: Supplementary file 3 [file Image_2.TIFF]

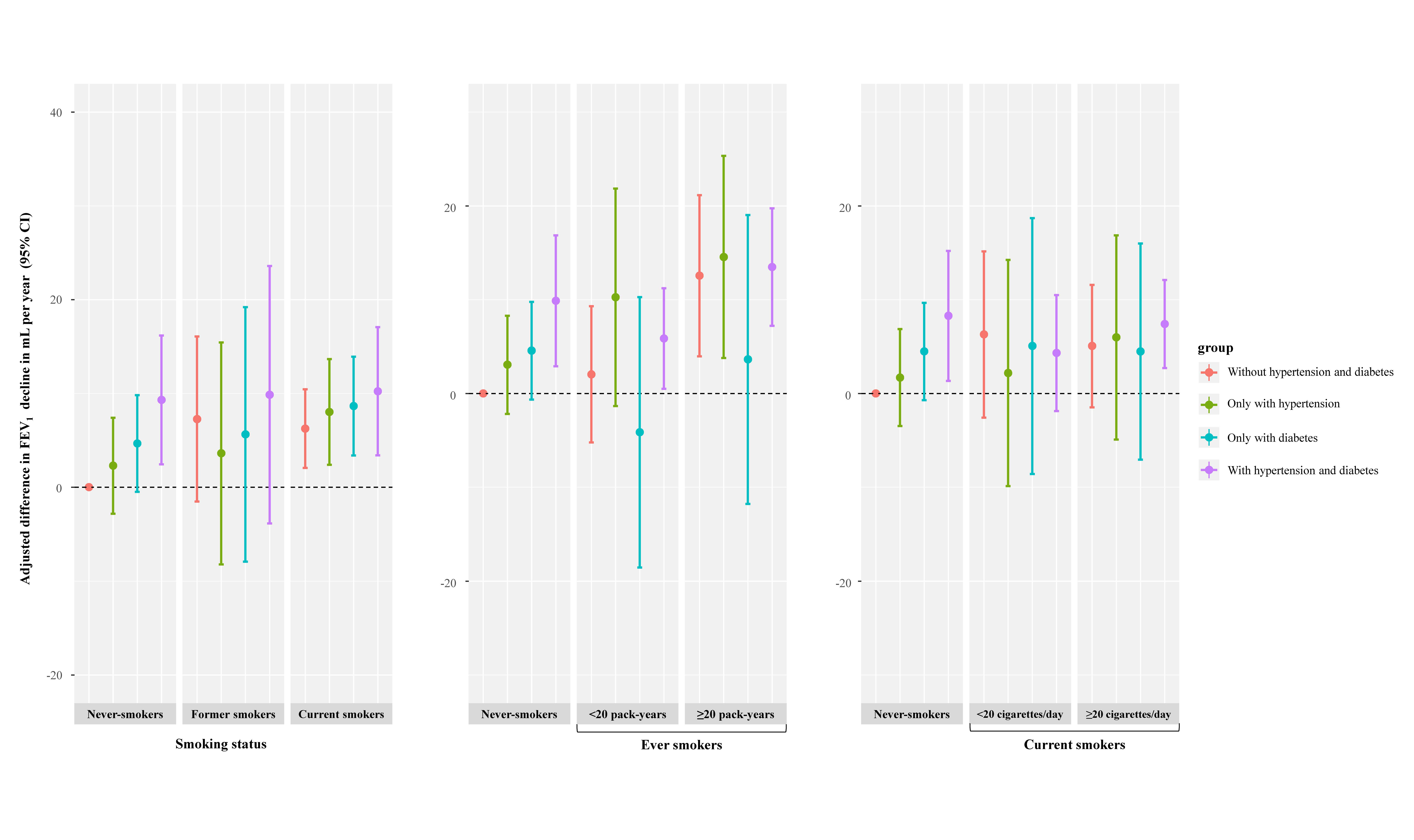

Supplement: Supplementary file 4 [file Image_3.TIF]
